# SCARF2 in the CSF of Asymptomatic subjects With a Parental History of AD and in the Cortex of Autopsy‐Confirmed Alzheimer’s Disease Cases

**DOI:** 10.1002/alz.086182

**Published:** 2025-01-03

**Authors:** Gabriel Aumont‐Rodrigue, Cynthia Picard, Anne Labonté, Judes Poirier, PREVENT‐AD research Group

**Affiliations:** ^1^ McGill University, Montreal, QC Canada; ^2^ Douglas Mental Health University Institute, Montreal, QC Canada; ^3^ Douglas Mental Health Research Centre, Montreal, QC Canada; ^4^ Centre for Studies on Prevention of Alzheimer’s Disease (StoP‐AD Centre), Montréal, QC Canada; ^5^ Douglas Mental Health University Institute, Montréal, QC Canada; ^6^ Centre for Studies on Prevention of Alzheimer’s Disease (StoP‐AD Centre), Douglas Mental Health University Institute, Montréal, QC Canada; ^7^ Centre for Studies on Prevention of Alzheimer’s disease (StoP‐AD Centre), Montreal, QC Canada

## Abstract

**Background:**

Scavenger receptors (SR) are a group of receptors involved in the endocytosis of various ligands, such as modified LDL and soluble β‐amyloid, which connects them to Alzheimer’s disease (AD). SCARF2 (SREC‐II) is part of the SR family, but unlike other scavenger receptors, internalizes a low amount of modified LDL. Its main function revolves around the binding of Aβ (Vo *et al.* 2023). Other studies found SCARF2 to be a SCARF1 inhibitor (Ishii *et al.* 2002). This study compares gene expression and protein levels of SCARF2 in post‐mortem brain tissue of AD and control subjects, as well as in the CSF of participants with a family history of AD. The goal is to track the progression of AD using the expression pattern of SCARF2.

**Method:**

SCARF2 CSF protein levels were assessed in the PREVENT‐AD (PRe‐symptomatic EValuation of Experimental or Novel Treatments for Alzheimer’s Disease) cohort which is composed of asymptomatic subjects with parental history of AD. SCARF2 has been measured with a primer extension assay using Olink technology. ELISA immunoassay has been used to quantify SCARF2 protein levels in frontal cortex homogenates of 86 brains from the Douglas Bell Canada Brain Bank (DBCBB). mRNA prevalence was measured using Gene Chip Clariom D human microarray. All statistical analyses were performed using JMP pro 17 software.

**Result:**

SCARF2 Protein levels, but not mRNA, are significantly lower in brains with AD (p<0.001). SCARF2 protein display a strong negative association with plaques bound Aβ, despite being a well‐established Aβ receptor (R^2^ = 0.277, p<0.0001). Interestingly, CSF protein levels correlate positively with AD biomarkers such as total and phosphorylated(181) tau (R^2^ = 0.235, p<0.0001; R^2^ = 0.288, p<0.0001) and Aβ to a lesser extent (R^2^ = 0.045, p = 0.0318).

**Conclusion:**

Our study unveils a distinctive role for SCARF2 in AD pathogenesis. Despite being a known Aβ receptor, SCARF2 exhibits lower cortical protein levels in AD brains, inversely linked to plaques bound Aβ. In contrast, elevated CSF SCARF2 protein levels correlate positively with CSF AD biomarkers in the presymptomatic phase of the disease; suggesting differential response to amyloid and tau deposition. These findings shed light on the intricate interplay of SCARF2 in AD pathophysiology, offering potential avenues for further research.

